# Hydrogen Sulfide Improves Vascular Calcification in Rats by Inhibiting Endoplasmic Reticulum Stress

**DOI:** 10.1155/2016/9095242

**Published:** 2016-02-28

**Authors:** Rui Yang, Xu Teng, Hui Li, Hong-Mei Xue, Qi Guo, Lin Xiao, Yu-Ming Wu

**Affiliations:** ^1^Department of Physiology, Institute of Basic Medicine, Hebei Medical University, Shijiazhuang 050017, China; ^2^Hebei Key Lab of Laboratory Animal Science, Hebei Medical University, Shijiazhuang 050017, China; ^3^Hebei Collaborative Innovation Center for Cardio-Cerebrovascular Disease, Shijiazhuang 050017, China; ^4^Key Laboratory of Vascular Medicine of Hebei Province, Shijiazhuang 050017, China

## Abstract

In this study, the vitamin D_3_ plus nicotine (VDN) model of rats was used to prove that H_2_S alleviates vascular calcification (VC) and phenotype transformation of vascular smooth muscle cells (VSMC). Besides, H_2_S can also inhibit endoplasmic reticulum stress (ERS) of calcified aortic tissues. The effect of H_2_S on alleviating VC and phenotype transformation of VSMC can be blocked by TM, while PBA also alleviated VC and phenotype transformation of VSMC that was similar to the effect of H_2_S. These results suggest that H_2_S may alleviate rat aorta VC by inhibiting ERS, providing new target and perspective for prevention and treatment of VC.

## 1. Introduction

Vascular calcification (VC) refers to the abnormal deposition of calcium and phosphorus minerals on the walls of blood vessels. The phenomenon of VC is common in atherosclerosis, hypertension, diabetic vascular disease, aging, and chronic kidney disease, and an important risk factor of cardiocerebrovascular diseases and related deaths [1]. There has been no clinical therapy specifically developed to treat VC.

The deposition of calcium mineral in VC was initially considered to occur passively. However, according to recent studies VC is actually an active biological process similar to bone development that is highly regulated, preventable, and can be reversed. In particular, the development and progression of VC involve the transformation of vascular smooth muscle cells (VSMCs) from a contractile to an osteoblast-like phenotype [2–5]. An agent that could prevent this transformation would have great guiding significance in research and potential for clinical application.

Recent studies have revealed that paracrine/autocrine vasoactive factors such as cardiovascular peptides [6], cytokines, and gaseous molecules [7] have an important regulatory role in the development and progression of VC. Hydrogen sulfide (H_2_S) is newly identified as a gaseous signaling molecule after discovery of nitric oxide and carbon monoxide. H_2_S affects the cardiovascular system in multiple ways, by dilating blood vessels, lowering blood pressure [8], and regulating the apoptosis and proliferation of VSMCs. Recent studies have suggested that H_2_S also inhibits VC and the phenotype transformation of VSMCs [9]. However, the specific mechanism is unknown. The thorough investigation of the mechanism of the ameliorative effect of H_2_S on VC should contribute to the transformation from bench to bedside and be useful to discover new target and strategy for treatment of VC.

The endoplasmic reticulum is the largest intracellular organelle of the cell. A disturbance of homeostasis in the endoplasmic reticulum leads to a series of cellular responses cumulatively known as endoplasmic reticulum stress (ERS). ERS has been implicated in the development and progression of a variety of cardiovascular diseases, and the development and progression of VC. The apoptosis of VSMCs that is caused by ERS promotes VC, and also the phenotype transformation of VSMCs [10–12]. In addition, recent studies have suggested that H_2_S may exert a protective effect on the cardiovascular system by inhibiting ERS [13–15]. Therefore, we hypothesized that H_2_S may alleviate VC by inhibiting ERS.

## 2. Materials and Methods

### 2.1. Rats and Reagents

All animal procedures complied with the Animal Management Rule of the Ministry of Health, People's Republic of China (documentation number 55, 2001) and the Care and Use of Laboratory Animals published by the United States National Institutes of Health (NIH Publication number 85-23, revised 1996) and approved by the Animal Care Committee of Hebei Medical University.

Male Sprague-Dawley rats (180–200 g) were supplied by the Animal Center of Hebei Medical University (Shijiazhuang, China) and housed under standard conditions (room temperature 20 ± 8°C, humidity 60 ± 10%, lights from 600 to 1800 h). They were freely supplied with standard rodent chow and water.

Tunicamycin (Tm) was from Cayman Chemical (Ann Arbor, USA). The alkaline phosphatase (ALP) and calcium detection kit was from BioSino Biotechnology and Science (Beijing, CN). Antibodies against calponin, RUNX2 (runt-related transcription factor 2), and GRP78 (78-kDa glucose-regulated protein) were from Epitomic (Burlingame, USA). Antibodies against SM22*α*, BMP2 (bone morphogenetic protein 2), active caspase-12, and beta-actin were from GeneTex (Irvine, USA). Antibodies against CHOP (C/EBP homologous protein) were from Affinity Biosciences (Cincinnati, USA). The secondary antibodies were from Kirkegaard & Perry Laboratories (Gaithersburg, USA). An enhanced chemiluminescence (ECL) kit was from MultiSciences Biotech (Hangzhou, CN). Other chemicals and reagents were of analytical grade.

### 2.2. The Model of VC* In Vivo*

Male Sprague-Dawley rats (180–200 g) were randomly divided into 7 groups (*n* = 6, each) as follows: the control; vitamin D_3_ plus nicotine (VDN); VDN + sodium hydrosulfide (NaHS); VDN + tunicamycin (Tm); VDN + Tm + NaHS; VDN + 4-phenylbutyric acid (PBA); and VDN + PBA + NaHS. In these groups, VDN treatment imposed calcification and therefore the VC model [16], NaHS acted as an H_2_S donor, Tm acted as an ERS agonist, and PBA as an ERS inhibitor.

The rats in the noncontrol groups were given vitamin D_3_ (300 000 IU/kg, intramuscularly) simultaneously with nicotine (25 mg/kg in 5 mL peanut oil, intragastrically) at 08:00 hours on the first day. The nicotine administration was repeated at 2000 hours. On days 2 and 15, the rats were retreated with vitamin D_3_.

In the rat groups that received NaHS (i.e., VDN + NaHS, VDN + Tm + NaHS, and VDN + PBA + NaHS), the NaHS was injected daily (56 *μ*mol/kg) during the 28 days of nicotine treatment. In the rat groups that received Tm (VDN + Tm and VDN + Tm + NaHS), Tm was injected daily (intraperitoneal, 5 *μ*g/kg) during the same period. In the rat groups that received PBA (VDN + PBA and VDN + PBA + NaHS), PBA was injected daily (intraperitoneal, 50 mg per rat) during the same period. Rats in the control group received normal saline intramuscularly (rather than vitamin D_3_) and 2 gavages of peanut oil (5 mL/kg, i.e., without nicotine) during the same period. The rats of the control and VDN groups were injected with saline vehicle (0.2 mL/100 g) per day, at a volume similar to the volumes of NaHS, Tm, and/or PBA injected into the rats of the other groups, during the same period. The aortic tissues of all the rats were harvested on day 28, as described below.

### 2.3. Blood Pressure Measurement

At the end of the 28-day experimental period, the rats were anesthetized with urethane (intraperitoneal, 1 g/kg). A cannula was inserted into the right carotid artery to measure blood pressure. Systolic blood pressure was measured using a pressure transducer (BL420F-Powerlab, TaiMeng, Chengdu, China). After measurement, the blood and aortas were collected, and the aortas were stripped of intima and adventitia and treated as described below.

### 2.4. Hematoxylin and Eosin (H&E) Staining

To quantify vascular medial calcification, the aortic roots (from the aortic opening to 0.5 cm from the opening section of the aorta) were separated and stored in 4% paraformaldehyde for H&E histopathological staining.

### 2.5. Quantification of Calcium Content and ALP Activity in Aortas

Calcium levels were determined by colorimetry through a reaction with o-cresolphthalein complexone. ALP activity was measured using an ALP Colorimetric assay kit, in accordance with the kit's instructions.

### 2.6. Western Blot Analysis

Aortas were homogenized in lysis buffer. Equal amounts of protein samples were loaded on 10% or 12% sodium dodecyl sulfate gels and then transferred to a nitrocellulose membrane. Nonspecific proteins were blocked with 5% nonfat dried milk for 1 h. Membranes were incubated serially with the primary antibodies overnight at 4°C, and with secondary antibody (horseradish peroxidase-conjugated anti-goat or anti-rabbit IgG) for 1 h. The reaction was visualized by enhanced chemiluminescence, and an autoradiograph was scanned. Protein concentrations were analyzed using NIH Image software and normalized to that of *β*-actin. All experiments were repeated at least 3 times.

### 2.7. Measurement of Plasma H_2_S Levels

Whole blood sample was collected, anticoagulated, and centrifuged at 3000 rpm for 15 min to obtain the plasma. H_2_S levels in plasma were measured as Shen et al. reported [17]. H_2_S levels were calculated using a standard curve generated from a sodium sulfide solution (0–100 *μ*M).

### 2.8. Statistical Analyses

Statistical analyses were performed using GraphPad software (GraphPad Prism v5.00 for Windows; GraphPad Software, San Diego, CA, USA). Comparisons between 2 groups were conducted using the unpaired Student's *t*-test. Comparisons among ≥3 groups were analyzed by one-way analysis of variance and then by Newman-Keuls test. Data are expressed as mean ± standard deviation. *P* < 0.05 was considered statistically significant.

## 3. Results

### 3.1. Comparison of VDN and Control Rats

**Figure 1:**
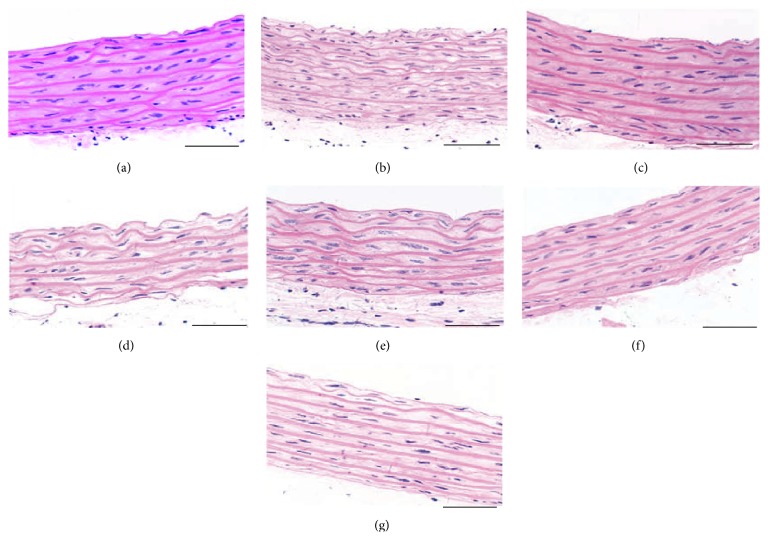
H&E staining of the aorta in rats (400x). (a) Control group. (b) VDN group. (c) VDN + NaHS group. (d) VDN + Tm group. (e) VDN + Tm + NaHS group. (f) VDN + PBA group. (g) VDN + PBA + NaHS group. Scale bar is 50 *μ*m.

**Figure 2:**
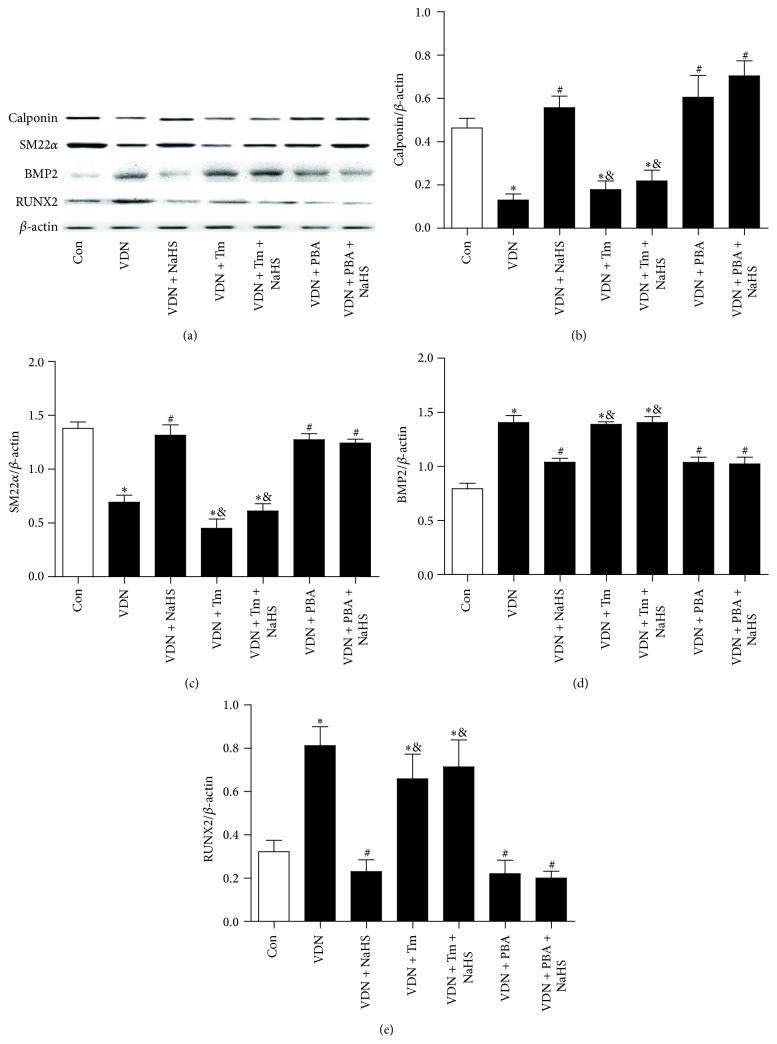
Tm blocks the effect of H_2_S on transformation in VSMCs in calcified aorta. (a) Representative protein levels of calponin, SM22*α*, BMP2, RUNX2, and *β*-actin; (b–e) quantitative analysis of protein levels of calponin, SM22*α*, BMP2, and RUNX2. ^*∗*^ *P* < 0.05 cf. control group; ^#^ *P* < 0.05 cf. VDN group; ^&^ *P* < 0.05 cf. VDN + NaHS group.

In the VDN (nontreated VC model) rat group, the mean systolic blood pressure, ALP activity, and calcium content of aortal tissues were significantly higher relative to that of the control rats (Table 1). Furthermore, in the VDN group, H&E staining revealed thickening and structural disorder of vascular elastic fibers (Figure 1), and the Western blot results showed that the protein levels of SM22*α* and calponin, that is, molecular markers of a VSMC contractile phenotype, were significantly lower than that of the controls. Conversely, in the VDN group the protein levels of BMP2 and RUNX2, which indicate a VSMC osteoblast-like phenotype, were significantly higher than that of the controls (Figure 2).

**Figure 3:**
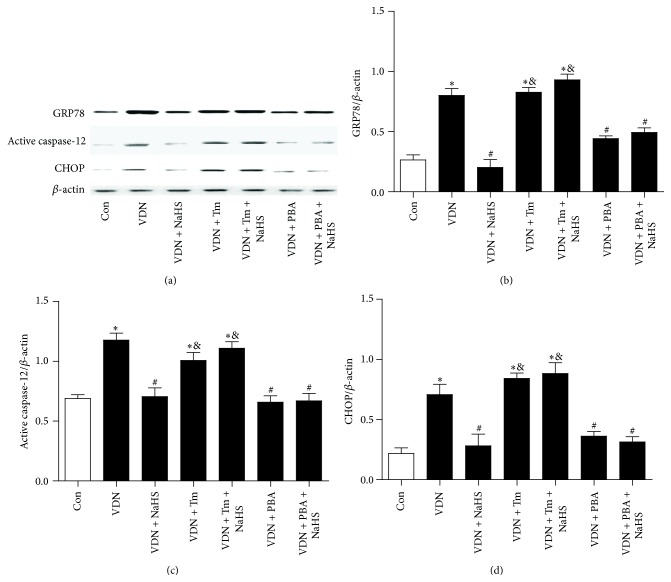
Tm blocks the effect of H_2_S on activation of ERS in calcified aorta. (a) Representative protein levels of GRP78, active caspase-12, CHOP, and *β*-actin; (b–d) quantitative analysis of protein levels of GRP78, active caspase-12, and CHOP. ^*∗*^ *P* < 0.05 cf. control group; ^#^ *P* < 0.05 cf. VDN group; ^&^ *P* < 0.05 cf. VDN + H_2_S group.

Compared with the control rats, in the VDN rats the protein levels of the ERS markers GRP78, active caspase-12, and CHOP, were all significantly higher (Figure 3).

### 3.2. Comparison of VDN + NaHS and VDN Rats

In the VDN + NaHS group, relative to the VDN rats the NaHS treatment was associated with higher systolic blood pressure and aortal tissue ALP activity, and calcium content was lower (Table 1). Furthermore, relative to the VDN rats, rats in the VDN + NaHS group appeared to have less thickening and structural disorder of vascular elastic fibers (Figure 1), higher protein levels of SM22*α* and calponin, and lower protein levels of BMP2 and RUNX2 (Figure 2).

Compared with the VDN rats, the VDN + NaHS rats had lower levels of the ERS markers GRP78, active caspase-12, and CHOP (Figure 3).

### 3.3. ERS Involved in the Ameliorated Effect of NaHS on VC in VDN Rats

The ERS inducer Tm and the ERS inhibitor PBA were used to investigate the role of ERS in VC. Tm could block the ameliorated effect of NaHS on VDN rats. Tm increased the ALP activity and calcium content (Table 1), downregulated the protein level of SM22*α* and calponin, and upregulated the protein level of BMP2 and RUNX2 in aorta of VDN rats with NaHS treatment (Figure 2). The protein level of GRP78, active caspase-12, and CHOP was also induced by Tm (Figure 3). However, there was no difference between VDN rats and VDN + Tm rats.

The effect of PBA on VC in VDN rats was similar as that of NaHS. PBA treatment also could decrease the ALP activity and calcium content in calcified aorta (Table 1). In VDN plus PBA rats, the downregulation of SM22*α* and calponin was reversed, and the upregulation of BMP2 and RUNX2 was simultaneously decreased (Figure 2). The increased protein level of GPR78, active caspase-12, and CHOP in calcified aorta was inhibited by PBA treatment (Figure 3). There was no synergistic effect of NaHS plus PBA on VDN rats.

### 3.4. Regulation of Akt Signaling Pathway in VC

**Figure 4:**
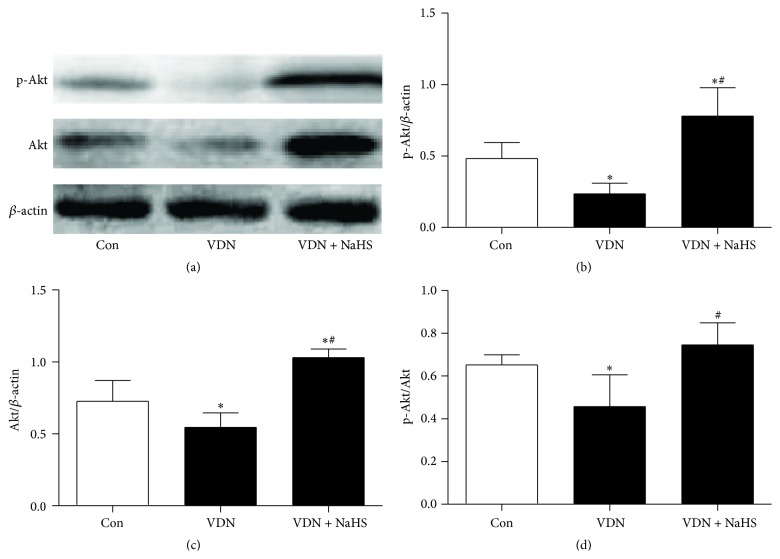
Regulation of Akt signaling pathway in aorta. (a) Representative protein levels of phospho-Akt, Akt, and *β*-actin; (b–d) quantitative analysis of protein levels of phospho-Akt and Akt. ^*∗*^ *P* < 0.05 cf. control group; ^#^ *P* < 0.05 cf. VDN group.

Compared with the control rats, the protein levels of Akt and phospho-Akt were both lower in the VDN rats. NaHS treatment significantly reversed the downregulation of Akt and phospho-Akt (Figure 4).

### 3.5. Protein Levels of Cystathionine *γ*-Lyase (CSE) in the Aorta and Plasma Levels of H_2_S

**Figure 5:**
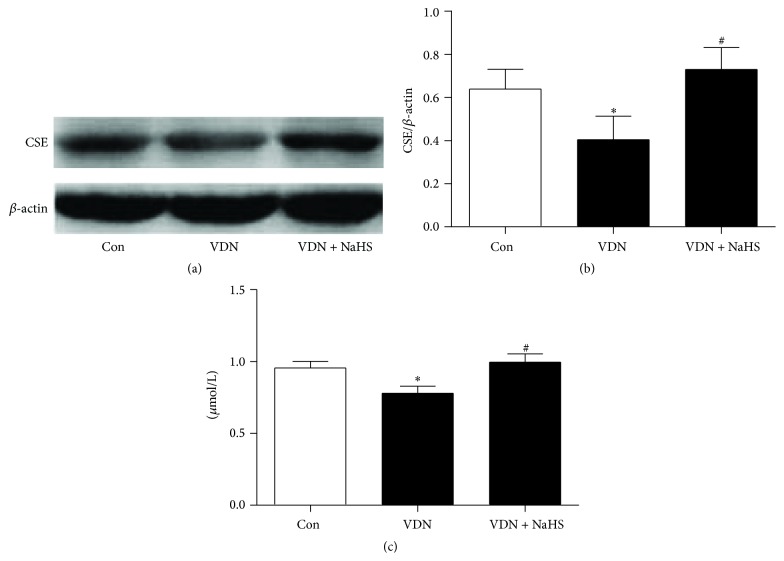
Protein levels of CSE in aorta and plasma levels of H_2_S. (a) Representative protein levels of CSE and *β*-actin; (b) quantitative analysis of protein levels of CSE. ^*∗*^ *P* < 0.05 cf. control group; ^#^ *P* < 0.05 cf. VDN group.

In the VDN rats, the protein levels of CSE in the aorta and plasma levels of H_2_S were lower than that in the control rats. NaHS treatment could significantly increase the protein levels of CSE and plasma levels of H_2_S in VDN rats (Figure 5).

## 4. Discussion

**Figure 6:**
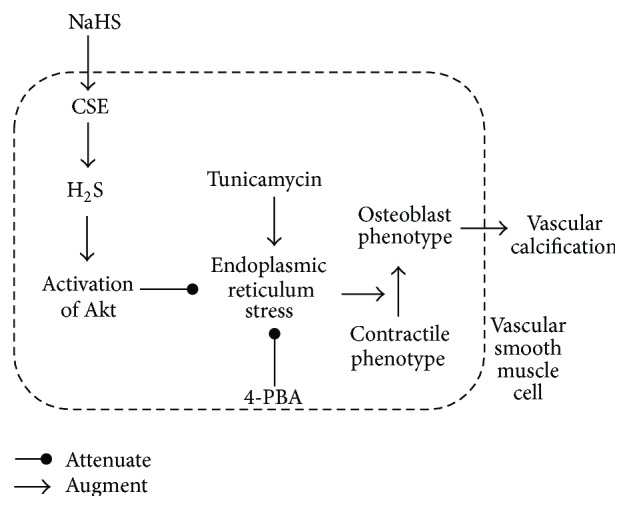
Schematic representation of the ameliorative effect of NaHS on VC.

In this study, a rat model of VC was established using the VDN method, to investigate the effect of H_2_S on VC and the phenotype transformation of VSMCs. In addition, H_2_S can also inhibit ERS of calcified aortic tissues. The effect of H_2_S on alleviating VC and the phenotype transformation of VSMCs can be blocked by Tm. PBA also alleviated VC and phenotype transformation of VSMCs, similar to the effect of H_2_S. Activation of Akt signaling may be involved in the ameliorated effect of H_2_S on ERS and VC. Exogenous NaHS treatment upregulated the protein expressions of aortic CSE and plasma levels of H_2_S in VDN rats. These results suggest that H_2_S may alleviate VC of the rat aorta by inhibiting ERS. Thus, H_2_S may be a new target for research of the prevention and treatment of VC (Figure 6).

It was initially assumed that VC is a passive process, resulting from disturbance of calcium/phosphorus metabolism and consequent deposition of these minerals. However, a major breakthrough in recent studies on the mechanism of VC revealed that VC is actually an active biological process similar to bone development, which is highly regulated, preventable, and can be reversed. The transformation of VSMCs from a contractile to osteoblast-like phenotype has been observed in both the vessels of patients with hypertension, diabetes, or chronic renal failure, and in the rat VC model established with a high-calcium, high-phosphorus, and high vitamin D_3_ and nicotine diet [18, 19]. In the present study, the calcium content and ALP activity of the VDN group were significantly higher than that of the control group. Western blot analysis showed that levels of the proteins that indicate a contractile phenotype (SM22*α* and calponin-1) were significantly lower in the VDN group relative to that of the control, while levels of the proteins associated with an osteoblast-like phenotype (RUNX2 and BMP2) were significantly higher. H&E staining revealed that, compared with the control group, the aortic elastic plate of the VDN group was significantly thicker and more disordered, with breakage of the elastic fibers. These results indicated that construction of the rat VC model was successful and confirmed the transformation of VSMCs from a contractile to osteoblast-like phenotype during VC.

H_2_S is a newly identified gaseous signaling molecule after nitric oxide and carbon monoxide. H_2_S has a rotten egg-like odor and is toxic, by inhibiting cytochrome C oxidase [20].* In vitro* studies have shown that H_2_S can reduce extracellular calcium deposition and inhibit expression of the genes responsible for the osteoblast-like transformation of VSMCs. In addition, H_2_S also inhibits upregulation of the sodium-phosphate cotransporter Pit-1, induced by uptake of phosphate and phosphoric acid. CSE inhibitor blocks these effects [11]. These results suggest that H_2_S strongly inhibits phosphate-induced VSMC calcification and osteoblast-like differentiation.

*In vivo* and* in vitro* experiments both indicate that H_2_S inhibits the phenotype transformation of VSMCs and alleviates VC [9]. In the present experiment, the calcium content of the aorta and ALP activity of the VDN group were both higher compared with that of the control group, while NaHS treatment significantly alleviated such increase. H&E staining revealed thickening and structural disorder of the vascular elastic fibers in the VDN group, and NaHS treatment significantly alleviated these changes. In addition, NaHS upregulated levels of protein related to the contractile phenotype of the aortic VSMCs, while inhibiting levels of proteins responsible for osteoblast-like phenotype. These results further show that H_2_S inhibits phenotype transformation in VSMCs and alleviates VC.

Any stimulus that interferes with endoplasmic reticulum function (such as hypoxia, oxidative stress, nutrition deficiency, and calcium homeostasis disturbance) may lead to accumulation of unfolded or misfolded protein in the endoplasmic reticulum, leading to ERS. Studies have indicated that apoptosis of VSMCs is an important aspect of VC, while ERS-induced apoptosis is obviously active in the calcified rat aorta [20–22]. Inhibition of ERS-induced VSMC apoptosis significantly alleviates development of VC [23–25]. Bone morphogenetic protein (BMP) signaling is a key aspect of VC that has an important role in osteoblast-like differentiation of VSMCs. It has been reported that BMP2 promotes VSMC calcification through induction of oxidative stress and ERS, and subsequently production of XBP1 (X-box binding protein 1), which binds to the Runx2 (runt-related transcription factor 2) promoter and promotes Runx2 expression [26]. In the present study, we also found that GRP78, CHOP, and active caspase-12 levels were significantly elevated in the calcified rat aorta, while the ERS inhibitor significantly alleviated VC. This indicated that ERS participates in the development of VC. NaHS inhibited ERS in the calcified rat aorta, and the ERS agonist blocked this effect of NaHS. These results suggest that H_2_S may alleviate VC by inhibiting ERS.

H_2_S putatively activates Akt signaling [27–30], and activation of the Akt signaling pathway has a crucial role in counteracting ERS [31–33]. Several factors could inhibit ERS by activating Akt signaling, resulting in a protective effect on the cardiovascular system [34]. Our present results showed that the protein levels of phospho-Akt and Akt were both upregulated by NaHS treatment. This suggests that H_2_S ameliorated ERS through activation of the Akt signaling pathway.

H_2_S is synthesized endogenously by the catalysis of 3 enzymes: CSE, cystathionine-*β*-synthase, and 3-mercaptopyruvate sulfurtransferase. CSE has a crucial role in maintaining cardiovascular homeostasis. Wu et al. [6] and our research team have both demonstrated that the expression and activity of CSE were reduced in the calcified aorta, which resulted in reduction of endogenous H_2_S. We have further shown that NaHS treatment upregulated the protein levels of CSE. The stimulation effect of exogenous H_2_S treatment on the production of endogenous H_2_S is in accord with other published articles [35, 36].

## 5. Conclusion

In this study, using a rat model of VC, we showed that H_2_S significantly alleviates VC, probably by inhibiting ERS. This study provides new strategies and a target for prevention and treatment of VC.

## Figures and Tables

**Table 1:** General characteristics and vascular total calcium and plasma and aortic ALP.

	SBP	Calcium	ALP	ALP
		Aorta, mmol/g DW	Plasma, IU/L	Aorta, IU/g protein
Control	118.1 ± 3.7	6.11 ± 2.21	135.4 ± 18.2	250.20 ± 60.22
VDN	155.8 ± 5.1^a^	51.38 ± 5.12^a^	349.4 ± 31.6^a^	462.42 ± 61.17^a^
VDN + NaHS	114.3 ± 5.3^b^	13.14 ± 8.56^b^	174.4 ± 23.6^b^	193.52 ± 23.34^b^
VDN + Tm	151.7 ± 2.6^a,c^	60.56 ± 10.01^a,c^	361.2 ± 26.8^a,c^	510.46 ± 41.10^a,c^
VDN + Tm + NaHS	150.0 ± 4.9^a,c^	55.56 ± 6.34^a,c^	340.6 ± 18.0^a,c^	490.20 ± 26.48^a,c^
VDN + PBA	110.3 ± 3.3^b^	14.01 ± 5.78^b^	125.4 ± 31.2^b^	221.56 ± 22.46^b^
VDN + PBA + NaHS	108 ± 9.9^b^	13.89 ± 8.02^b^	133.0 ± 28.0^b^	216.08 ± 31.64^b^

^a^ *P* < 0.05 cf. control group; ^b^ *P* < 0.05 cf. VDN; ^c^ *P* < 0.05 cf. VDN + H_2_S group.

SBP, systolic blood pressure.
